# The Positive Effects of Yerba Maté (*Ilex paraguariensis*) in Obesity

**DOI:** 10.3390/nu7020730

**Published:** 2015-01-22

**Authors:** Alessandra Gambero, Marcelo L. Ribeiro

**Affiliations:** Laboratory of Microbiology and Molecular Biology, Clinical Pharmacology and Gastroenterology Unit, Sao Francisco University Medical School, Av São Francisco de Assis 218, Bragança Paulista, SP 12916-900, Brazil; E-Mail: alessandra.gambero@usf.edu.br

**Keywords:** yerba maté, *Ilex paraguariensis*, obesity, adipogenesis, inflammation

## Abstract

The prevalence of obesity has increased worldwide over the past three decades. Global anti-obesity strategies focus on dietary and lifestyle modifications to slow the development of obesity. Research in the nutrition field has recently aroused considerable interest based on the potential of natural products to counteract obesity. Several studies have identified yerba maté (*Ilex paraguariensis*) as an excellent candidate. In this review, we evaluated the impact of yerba maté on obesity and obesity-related inflammation. Cellular studies demonstrate that yerba maté suppresses adipocyte differentiation and triglyceride accumulation and reduces inflammation. Animal studies show that yerba maté modulates signaling pathways that regulate adipogenesis, antioxidant, anti-inflammatory and insulin signaling responses. In summary, the data presented here showed that the use of yerba maté might be useful against obesity, improving the lipid parameters in humans and animal models. In addition, yerba maté modulates the expression of genes that are changed in the obese state and restores them to more normal levels of expression. In doing so, it addresses several of the abnormal and disease-causing factors associated with obesity. Protective and ameliorative effects on insulin resistance were also observed. Thus, as a general conclusion, it seems that yerba maté beverages and supplements might be helpful in the battle against obesity.

## 1. Introduction

The prevalence of obesity has increased worldwide. Obesity is a complex condition involving social, biological and psychosocial factors. A sedentary lifestyle and a high-calorie diet seem to be the most important factors in the development of obesity. Co-morbidities associated with obesity are serious and include several metabolic disorders, such as diabetes type 2 and atherosclerosis. Global anti-obesity strategies focus on dietary and lifestyle modifications to slow the development of obesity. Research in the nutrition field has recently aroused considerable interest based on the potential of natural products to counteract obesity. Several studies have identified yerba maté (*Ilex paraguariensis* A.St.-Hil) as an excellent candidate. In this review, we evaluated the *in vitro* and *in vivo* impact of yerba maté on obesity and obesity-related inflammation.

## 2. Background on Obesity and Its Mechanisms

The prevalence of obesity is a major public health concern, because of the associated weight-related diseases that result in significant morbidity and mortality and reduced quality of life. The energy balance defect that causes obesity and visceral adiposity is serious and predisposes individuals to complications, such as atherosclerosis, hepatic steatosis and type 2 diabetes [1]. The increasing incidence of obesity suggests that this epidemic will continue to grow [2]. A low-grade inflammatory process in adipose tissue has been identified as key in the development of obesity-associated pathologies, such as type 2 diabetes and cardiovascular diseases. In addition to adipose tissue’s main role in releasing fatty acids to be used as energy substrates, this tissue is an active endocrine organ, secreting several hormones and signaling substances with a number of biological functions. Satiety and appetite control, glucose and lipid metabolism, blood pressure regulation and inflammation and immune modulation are altered by adipose tissue-derived substances.

Two important observations contributed to a change in the point of view regarding adipose tissue biology. The first was the discovery of leptin, an important hypothalamic satiety signal [3], in 1994 [4], and the subsequent data describing this hormone’s proinflammatory actions [5]. The second was the description of adipocyte synthesis and the release of tumor necrosis factor (TNF)-α, a classical proinflammatory substance [6].

The exact mechanism by which adipose tissue develops local inflammation during obesity is not fully understood. The mediators and mechanisms involved are complex and multifactorial. Adipose tissue contains not only adipocytes, but also adipocyte precursors, nerve terminals, blood vessels and immune cells, collectively named the stromal vascular fraction (SVF). In 2003, Weisberg described that approximately 40% of SVF cells from visceral adipose tissue in obese mice are macrophages compared to only 10% of SVF cells in lean controls [7]. In addition, infiltrated macrophages in adipose tissue from lean mice are different from those in adipose tissue from obese mice. Obesity induces not only macrophage infiltration, but also alterations in the macrophages’ phenotype. The M2:M1 macrophage ratio is reduced in adipose tissue of obese mice [8]. Human adipose tissue macrophages also present M1 characteristics [9]. M2 macrophages produce anti-inflammatory cytokines, such as interleukin (IL)-10, and have important functions in repair and remodelling, whereas M1 macrophages produce proinflammatory cytokines, such as TNF-α and IL-1β, and have phagocytic and bactericidal functions [10]. Alterations in free fatty acid (FFA) concentrations could provide a chemotactic stimulus for macrophage infiltration through arachidonic acid products or the release of chemokines, such as monocyte chemoattractant protein-1 (MCP-1) [11,12]. Another hypothesis is that adipose tissue hypertrophy leads to hypoxia, resulting in local inflammation. Hypertrophied adipocytes can become as large as 150 to 200 μM in diameter, and oxygen diffusion is impaired under this condition. Local hypoxia and upregulation of hypoxia-activated genes, like hypoxia-inducible factor-1 (HIF-1α) and vascular endothelial growth factor (VEGF), have been described in adipose tissue from mice and humans in obesity [13,14]. In addition, adipocytes are very sensitive to hypoxia and respond with an increase in proinflammatory mediator production [13,15]. Macrophage infiltration in response to lipotoxicity or hypoxia initiates recruitment of additional inflammatory cells, mainly through MCP-1 release, and results in elevated local production of proinflammatory cytokines, such as TNF-α, IL-6 and IL-1β, by these cells. These cytokines activate inflammatory pathways, resulting in activation of Jun *N*-terminal kinase-1 (JNK-1) and κB kinase (IKKβ) inhibitor [16,17]. IKKβ disturbs insulin signaling through direct phosphorylation of insulin receptor substrate-1 (IRS-1) on serine residues or by phosphorylation of the nuclear factor-κB (IκB) inhibitor, which dissociates nuclear factor-κB (NF-κB), allowing this factor to translocate to the nucleus and activate inflammatory genes, such as TNF-α, IL-6 and MCP-1 [16,17,18]. In addition, the JNK-1 signaling pathway also results in serine phosphorylation of IRS-1 and inflammatory gene transcription via transcription factor activator protein 1 (AP1) [19].

Inflammatory mediators produced in adipose tissue decrease the capacity of preadipocytes to differentiate. Defective adipogenesis is related to a decrease in the expression of differentiation-related genes, such as peroxisome proliferator-activated receptor-γ2 (PPAR-γ2) and transcription factor CCAAT/enhancer-binding protein (C/EBP)-α [20]. Thiazolidinediones (TZDs), which are known to activate PPAR-γ, are able to improve insulin signaling and glucose uptake by adipose tissue, despite also resulting in side effects, such as obesity [21].

Regarding adipogenesis, it involves a series of sequential events, such as cell cycle arrest, clonal expansion and differentiation. These events require several genes, the process probably begins with the activation of C/EBP-α, C/EBP-β and C/EBP-δ and is followed by PPARγ activation, which acts directly on different genes associated with adipogenesis [22,23,24]. Several studies have shown that PPARγ is a major regulator of adipogenesis and that the maintenance of its expression is essential for the progression through the late stages of differentiation [25]. PPARγ2 is primarily expressed in adipose tissue and promotes the differentiation and proliferation of adipocytes, which are derived from fibroblasts, resulting in an increase in adiposity [26]. Furthermore, the activation of PPARγ results in the expression of several pro-adipogenic genes, including C/EBP-α [27]. Therefore, it has been suggested that the self-regulation of C/EBP-α and PPARγ is critical for maintaining adipocyte differentiation [28,29].

## 3. The Effects of Yerba Maté in Obesity

Diet is an important regulatory factor of the inflammatory response and is directly responsible for obesity development in most obese subjects. Dietary bioactive compounds, such as polyphenols and certain fatty acids, are reported to suppress both systemic and adipose tissue inflammation and potentially improve these obesity-associated metabolic disorders [30].

*Ilex paraguariensis* (*Aquifoliaceae*), known commonly as yerba maté, is a plant originally from the subtropical region of South America, including southern Brazil, northern Argentina, Paraguay and Uruguay [31]. The aqueous extract of yerba maté is mainly prepared as four different beverages: chimarrão, maté cocido, tererê and maté tea. Both chimarrão and tererê are made with dried and crushed green maté leaves. The first is prepared with hot water, and the second is prepared with cold water. Maté tea is prepared with roasted leaves and brewed as any other herbal tea. Maté cocido refers to green maté brewed as an herbal tea, usually commercialized in bags sold as maté tea [32].

Maté beverages have been reported to have various biological activities, which have been attributed to the high polyphenol content of yerba maté. Phenolic compounds have long been known to possess biological functions. In addition to polyphenols, such as flavonoids (quercetin and rutin) and phenolic acids (chlorogenic and caffeic acids), yerba maté is also rich in caffeine and saponins [32]. Recently published research has scientifically proven that yerba maté has important pharmacological properties, such as antioxidant activity [33,34,35,36,37,38], protective effects against induced DNA damage [35], vasodilation activity [39], inhibition of glycation and atherosclerosis [31,40,41,42], improvement in glucose tolerance [42,43,44], anti-inflammatory effects [45,46,47,48,49], chemopreventive properties [50,51,52,53], thermogenic effects [49,54], amelioration of insulin resistance [42,45,55,56,57] and anti-obesity effects [44,46,47,48,49,54,56,58,59,60,61,62,63]. In addition, recently, Bracesco *et al.* [64] published a very informative and comprehensive review in which translational studies, inflammation and lipid metabolism were updated. Thereby, for further information about the issues that are not addressed in this review, it is recommended to read it.

The interest in yerba maté for health promotion is relatively recent. In the mid-90s, the first scientific evidence was published demonstrating the *in vitro* and *in vivo* antioxidant activity of yerba maté [33,65]. Likewise, antioxidant activity has been observed for many other natural products. Some researchers have also focused efforts on understanding the role of yerba maté in the modulation of obesity and obesity-associated conditions.

In 2001, the first study to evaluate the anti-obesity role of yerba maté was conducted. In this clinical study, the authors demonstrated that an herbal preparation containing yerba maté (“YGD”, yerba maté; guarana, *Paullinia cupana* Kunth; and damiana, *Turnera diffusa* Willd) significantly delayed gastric emptying, reducing the time to perceived gastric fullness, and induced significant weight loss over 45 days in overweight patients [66]. Subsequently, YGD has been demonstrated to produce a robust acute effect on caloric intake and meal duration, suggesting that YGD strengthens within-meal satiation, an effect that may be mediated by the previously reported changes in gastric emptying [67]. Moreover, in an elegant study, de Morais *et al.* [68] demonstrated the hypocholesterolemic effects of yerba maté in healthy subjects with normo- or dyslipidemia. The authors observed, after 20 and 40 days of treatment, a significantly reduction on the levels of low-density lipoprotein-cholesterol (LDL-C), non-high density lipoprotein cholesterol (non-HDL-C), apolipoprotein B (apo B), the LDL-C/HDL-C ratio and increasing HDL-C. In addition, it was demonstrated that yerba maté produced additional LDL-C lowering in hypercholesterolemic subjects who were on stable statin therapy, which may reduce the risk for cardiovascular diseases (Table 1).

**Table 1:** *In vivo* effects of yerba maté on adipogenesis: human and animals studies.

Study	Type of Study	Population	Test Compounds (Daily Dosage)	Duration of Intake	Main Outcomes
Andersen and Fogh, 2001 [66]	Double-blind placebo-controlled parallel trial	Forty-seven healthy overweight (body mass index (BMI) range of 25.8 ± 30.4 kg/m^2^) volunteers.	Three tablets of YGD (112 mg yerba maté, 95 mg guarana and 36 mg damiana extract) before each main meal.	10 days and 45 days and weight maintenance over 12 months	YGD significantly increased gastric emptying time of 58 ± 15 min compared to 38 ± 7.6 min after placebo; significantly increased body weight reductions over 10 days (0.8 ± 0.05 kg after YGD compared with 0.3 ± 0.03 kg after placebo) and over 45 days (5.1 ± 0.5 kg after YGD compared to 0.3 ± 0.08 kg after placebo); treatment with YGD resulted in weight maintenance (73 kg at the beginning and 72.5 kg at the end of 12 months).
De Morais *et al.*, 2009 [68]	Single-blind controlled trial	One hundred and two volunteers (*n* = 36 male and 66 female; mean age = 48.4 ± 1.35 years). Divided into 3 groups: normolipidemic (*n* = 15), dyslipidemic (*n* = 57) and hypercholesterolemic subjects on long-term statin therapy (*n* = 30).	330 mL, 3-times/day of green or roasted yerba maté infusions.	40 days	Normolipidemic subject treated with yerba maté exhibited a significative reduction of 8.7% on LDL-cholesterol. Dyslipidemic individuals lowered LDL-cholesterol levels up to 8.6% and non-HDL cholesterol up to 6.5%. The apolipoprotein B level was reduced by 6.0%, and HDL-cholesterol was significantly increased by 4.4%. The yerba maté consumption by hypercholesterolemic individuals on statin therapy promoted an additional 13.1% reduction in LDL-C and increased HDL cholesterol by 6.2%.
Harrold *et al.*, 2013 [67]	Double blind, placebo-controlled, crossover	Fifty-eight healthy women, aged 18–65, with a BMI between 18.5 and 29.9 kg/m^2^.	Three tablets of YGD (112 mg yerba maté, 95 mg guarana and 36 mg damiana extract) and inulin-based soluble fermentable fiber (SSF; 5 g in 100 mL water), 3 tablets of YGD and water (100 mL), SFF and placebo (3 tablets) or water and placebo 15 min before lunch.	1 day	YGD and SFF significantly reduced food and energy intake (59.5 g, 16.3%; 112.4 kcal, 17.3%; and 31.9 g, 9.1%; 80 kcal, 11.7%, respectively) compared with conditions where they were absent. The lowest intake (gram and kcal) was observed in the YGD + SFF combination. In summary, YGD causes a robust short-term effect on caloric intake, an effect augmented by SFF.
Pang *et al.*, 2008 [58]	Experimental	Sprague-Dawley rats fed with high-fat diet (3 groups, *n* = 8 each).	High-fat diet (HFD) supplemented with maté (*ad libitum*).	8 weeks	Yerba maté extract significantly reduced the final body weight. It reversed the HFD-induced downregulation of the adipose tissue genes implicated in adipogenesis or thermogenesis. Significant decreases in the phospho-AMP-activated protein kinase (AMPK)/AMPK protein ratio were also observed.
Arçari *et al.*, 2009 [49]	Experimental	Swiss mice fed with high-fat diet (3 groups, *n* = 10 each).	1 g/kg of roasted yerba maté extract by oral route (once per day).	8 weeks	Yerba maté significantly inhibited the inflammation in adipose tissue induced by obesity, modulating several pro- and anti-inflammatory genes and reducing macrophage infiltration.
Martins *et al.*, 2010 [63]	Experimental	Swiss mice fed with high-fat diet (4 groups, *n* = 10 each).	1 and 2 g/kg of roasted yerba maté extract by oral route (once per day).	8 weeks	Yerba maté extract significantly reduced the final body weight. A reduction of total serum cholesterol and LDL-cholesterol levels was observed. Serum triglycerides were also significantly reduced. Yerba maté significantly reduced lipid accumulation in the liver (decreased by ~30%).
Arçari *et al.*, 2011 [45]	Experimental	Swiss mice fed with high-fat diet (3 groups, *n* = 10 each).	1 g/kg of roasted yerba maté extract by oral route (once per day).	8 weeks	Yerba maté significantly improved insulin resistance by restoring hepatic and muscle IRS-1 and AKT phosphorylation and by controlling adipose tissue inflammation associated with obesity.
Hussein *et al.*, 2011 [43]	Experimental	Male Tsumura Suzuki obese diabetic (TSOD) mice (3 groups, *n* = 5 each).	100 mg/kg of an aqueous extract of yerba maté by oral route (once per day).	7 weeks	Significantly ameliorated metabolic syndrome by improving peripheral insulin sensitivity and cellular glucose uptake and by modulating the level of circulating lipid metabolites and adiponectin.
Hussein *et al*., 2011 [61]	Experimental	DdY mice fed with high-fat diet.	Aqueous extract of maté by oral route (once per day).	3 weeks	Administration of yerba maté induced significant increases in GLP-1 levels and leptin levels, generating anorexic effects by direct induction of satiety.
Kang *et al*., 2012 [44]	Experimental	C57BL/6J mice fed with high-fat diet (5 groups, *n* = 5 each).	0.5, 1 or 2 g/kg of aqueous extract of yerba maté by oral route (once per day).	4 weeks	Yerba maté consumption significantly reduced the body weight, adiposity, adipocyte size and leptin release by adipose tissue. Maté-treated mice also presented a significant reduction in serum levels of triglycerides and cholesterol.
Pimentel *et al*., 2012 [46]	Experimental	Wistar rats fed with high-fat diet (3 groups, *n* = 7 each).	From 100 to 200 mg/kg of yerba maté extract by oral route (once per day).	8 weeks	Yerba maté significantly inhibited hypothalamic inflammation induced by obesity trough reducing the phosphorylation of hypothalamic IKK and NFκBp65 expression and significantly increasing the protein levels of IκBα and adiponectin receptor-1. Inflammation associated with obesity in liver and muscle was also significantly controlled by yerba maté.
Arçari *et al*., 2013 [57]	Experimental	Swiss mice fed with high-fat diet (3 groups, *n* = 10 each).	1 g/kg of roasted yerba maté extract by oral route (once per day).	8 weeks	Significantly improved insulin resistance by restoring hepatic FOXO1 nuclear translocation and upregulating gene expression of *Akt2, Irs1, Irs2, Pi3kca, Pi3kcg* and* Pdk1.*
Arçari *et al*., 2013 [59]	Experimental	Swiss mice fed with high-fat diet (3 groups, *n* = 10 each).	1 g/kg of roasted yerba maté extract by oral route (once per day).	8 weeks	Yerba maté extract significantly reduced the final body weight. It downregulated the expression of genes that regulate adipogenesis and upregulated the expression of genes related to the inhibition of adipogenesis.
Borges *et al*., 2013 [48]	Experimental	Wistar rats fed a with high-fat diet (4 groups, *n* = 8–12 each).	1 g/kg of roasted yerba maté extract by oral route (once per day).	4 weeks	The consumption of yerba maté promoted weight loss, attenuated the detrimental effects of HFD on adiposity and insulin sensitivity and decreased the blood levels of inflammatory biomarkers. Concerning peritoneal macrophages, maté decreased the mRNA production of *Il-6*, but did not influence the production of *Il-1β*, *Tnf-α* and nitric oxide.
Carmo *et al*., 2013 [47]	Experimental	Wistar rats fed a with high-fat diet (4 groups, *n* = 8–12 each).	1 g/kg of roasted yerba maté extract by oral route (once per day)	4 weeks	Significantly reduced body weight, body adiposity and cholesterol levels. Maté consumption reduced IL-1α, IL-6 and TNF-α production by bone marrow cells.
Gao *et al*., 2013 [69]	Experimental	Sprague-Dawley rats fed with high-fat diet (5 groups, *n* = 12 each).	1 2 and 4% yerba maté extract. The animals had free access to bottles containing the prepared infusion as the only available liquid source.	4 weeks	Yerba maté may regulate blood lipid and endothelial function in hyperlipidemia rats. The putative mechanism may include a reduction of endothelin and thromboxane A_2_ levels and an increase in nitric oxide and 6-keto-PGF1α levels in the blood, downregulating the expression of ICAM-1 (Intercellular Adhesion Molecule 1) protein and upregulating the expression of LDLR (LDL receptor) and SR-B1 (scavenger receptor class B member 1) genes, inhibiting the occurrence of atherosclerosis.
Lima *et al*., 2014 [56]	Experimental	Wistar rats primed by early weaning.	1 g/kg of yerba maté extract by oral route (once per day).	4 weeks	Yerba maté consumption significantly reduces body weight, adiposity and triglycerides levels in the blood.

In addition to human studies, in DdY mice fed with high-fat diet animal models, yerba maté has been suggested to promote satiety through various mechanisms, including induction and/or enhancement of intestinal glucagon-like peptide-1 (GLP-1), modulation of serum leptin levels and a possible direct central satiety-stimulatory effect [61]. Data obtained from experiments conducted in diet-induced obesity models have shown that yerba maté suppresses body weight gain and visceral fat accumulation and decreases serum levels of cholesterol, triglycerides, LDL cholesterol, glucose, insulin, pancreatic lipase and leptin [39,44,47,48,49,56,58,59]. Additionally, yerba maté reduces endothelin and thromboxane A_2_ levels and increases nitric oxide and 6-keto-PGF1α levels in the blood, inhibiting the occurrence of atherosclerosis [69]. It has been suggested that the high polyphenol content of yerba maté might be responsible for these observed results. In this sense, chlorogenic acid, the main polyphenol in yerba maté, is thought to modulate the activity of glucose-6-phosphatase, which is involved in glucose metabolism [70], and to reduce the risk of cardiovascular disease by decreasing LDL and cholesterol oxidation [71]. Additionally, it has been suggested that the hypolipidemic effects of yerba maté could be attributed, at least in part, to its saponin content [72] (Table 1).

**Figure 1:**
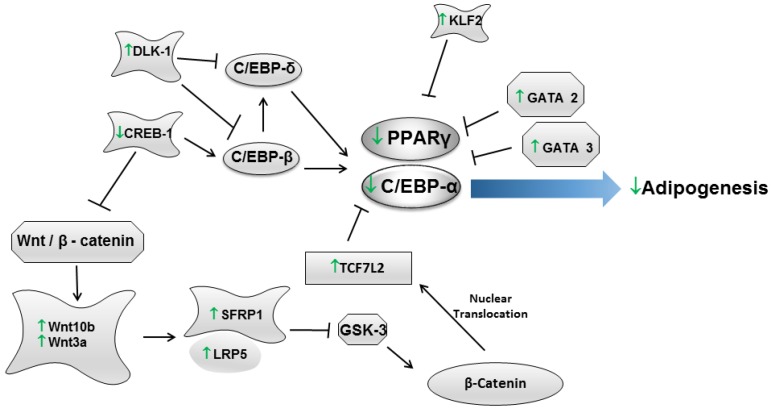
*In vivo* and *in vitro* effects of yerba maté in adipogenesis.

The molecular mechanisms by which yerba maté regulates obesity have also been studied. In this regard, several studies have been conducted in cellular models and in obese animals to evaluate the effects of yerba maté on several genes related to adipogenesis. Adipogenesis is the developmental process by which a multipotent mesenchymal stem cell differentiates into a mature adipocyte. This process involves a highly regulated and coordinated cascade of transcription factors, including members of the PPAR, C/EBP and sterol regulatory element-binding protein (SREBP) families, which together lead to the establishment of the differentiated state [21]. In this context, it has been observed that yerba maté modulates adipogenesis by regulating the gene expression levels of pro-adipogenic transcription factors, such as *Ppar-γ2* [49,58,59] and *C/ebp-α* [59], *in vivo* and *in vitro* [59,60]. C/EBP and *Ppar-γ2* expression depends on other genes that are also essential to adipogenesis, such as cAMP responsive element binding protein 1 (Creb1) and delta-like 1 homolog (*Drosophila*) (Dlk1); Arcari *et al.* [59] showed that yerba maté modulates the *in vivo* and *in vitro* expression of these genes, thus contributing directly to adipogenesis regulation (Figure 1, Table 1 and Table 2).

In addition, it has been shown that yerba maté regulates adipogenesis in a β-catenin-dependent manner [59]. The β-catenin-dependent signaling pathway is initiated by the binding of the wingless-type MMTV integration site family (WNT) 1, WNT3a and WNT10b to secreted frizzled-related protein (SFRP) 1 and SFRP5 and low density lipoprotein receptor-related protein (LRP) 5 and LRP6 receptors. The expression of WNT10b stabilizes β-catenin in the cytoplasm, thereby inhibiting adipogenesis. Data indicate that among the WNT proteins, WNT10b is the primary adipogenesis regulator, and WNT1 and WNT3a act synergistically [73]. The binding of WNT to its receptors (SFRP and LRP) inhibits glycogen synthase kinase (GSK)-3, resulting in the hypophosphorylation of β-catenin. β-Catenin translocates to the nucleus, where it binds to a TCF/LEF transcription factor, repressing the expression of C/EBPα and PPARγ and, therefore, inhibiting adipogenesis [24]. Arcari *et al*. [59] demonstrated that yerba maté modulates adipogenesis *in vitro* and *in vivo* via the WNT pathway by increasing the mRNA levels of *Wnt10B, Wnt3A, Sfrp1* and *Lrp5*, which favor the nuclear translocation of β-catenin, thereby increasing the expression of *Tcf7l2*. An increase in *Tcf7l2* (transcription factor 7-like 2 (T-cell specific, HMG-box)) could lead to the repression of C/ebpα and *Ppar-γ2*, thereby reducing adipogenesis. In addition, it has been shown that the GATA and Krüppel-like zinc finger (KLF) proteins also directly affect adipogenesis. It has been suggested that increased expression of GATA-2 and GATA-3 suppresses adipocyte differentiation through a direct repression of C/EBP-α and PPARγ [74]. Regarding KLF, it has been demonstrated that KLF2 inhibits adipogenesis by inhibiting PPARγ [75]. Arcari *et al*. [59] observed that yerba maté enhanced the gene expression of *Gata2, Gata3* and *Klf2*, which may contribute to the inhibition of adipogenesis via the PPARγ pathway (Figure 1, Table 1 and Table 2). Taking into account that yerba maté is rich in several bioactive compounds, it has been attempted to assess whether any of these compounds would have a greater effect in the inhibition of adipogenesis [59,60]. The results from these showed that synergism between these compounds might be responsible for the results observed in the yerba maté intervention group.

**Table 2:** *In vitro* effects of yerba maté on adipogenesis.

Study	Type of Cell (Origin)	Assay Employed	Tested Compounds (Concentration)	Main Outcomes
Martins *et al*., 2010 [63]	None *	Determination of lipase activity inhibitory action against porcine and human lipases and the influence of several olive oil emulsifying reagents.	0.5–5.0 mg/mL of roasted yerba maté.	Yerba maté significantly inhibited the enzyme activities in a dose-dependent way, and its inhibitory activity against both lipases reached a maximum at 3.0 mg/mL, corresponding to 9 mg of tea/g substrate. Kinetic results indicated that yerba maté competitively inhibited pancreatic lipase activity in a concentration-dependent manner with a half-maximal inhibitory concentration value of 1.5 mg yerba maté/mL (or 4.5 mg of yerba maté/g of substrate), whereas a drastic decrease in lipolytic activity (>80% that of the control) was observed in the presence of 3.0 mg yerba maté/mL.
Gosmann *et al*., 2012 [60]	3T3-L1 (*Mus musculus*)	Determination of phenolic compounds (Folin-Ciocalteu method), Oil Red O staining and gene expression.	Extracts of both fresh and dried maté leaves subjected to chromatography in order to obtain the saponin (20% yield) and the polyphenol extracts (40% yield).	Among the yerba maté samples, the polyphenol extract of fresh leaves exhibited a higher content of total phenols, followed by the polyphenol extract and the ethanol extract of dried leaves. Saponin extracts of both fresh and dried leaves exhibited lower contents of phenolic compounds. Regarding adipogenesis, the highest anti-adipogenic effect was detected in the polyphenol extract of dried leaves at 50 μg/mL, followed by the saponin extract of fresh leaves at 100 μg/mL and by the polyphenol extract of fresh leaves at 500 μg/mL. All assayed samples restrained the expression of the *PPARγ2, Lep, TNF-α and C/EBPα* genes.
Arcari *et al*., 2013 [59]	3T3-L1 (*Mus musculus*)	Oil Red O staining and gene expression (Mouse Adipogenesis RT^2^ Profiler™ PCR Array).	50, 250 or 500 µg/mL of roasted yerba maté, chlorogenic acid, quercetin, and rutin (Sigma-Aldrich).	Yerba maté extract and chlorogenic acid inhibited adipogenesis at a concentration of 50 µg/mL. Quercetin and rutin had inhibitory activity at the highest concentration. The PCR array revealed that yerba maté modulated the expression of 14 genes (belonging to PPARγ and WNT signaling pathways) that are associated with adipogenesis. In addition, the bioactive compounds also modulated the expression of adipogenesis-associated genes. However, fewer genes were regulated by chlorogenic acid, quercetin and rutin than by yerba maté. The authors claim that the synergism between these compounds might be responsible for the results observed.

* Pancreatic lipase activity was based on the amount of free fatty acids liberated from emulsified olive oil (using human and porcine lipases that were commercially available).

Furthermore, besides the effects on adipogenesis regulators, yerba maté has been demonstrated to act on the expression of genes related to thermogenesis. Pang *et al.* [58] demonstrated that yerba maté can have a protective effect against obesity in a rodent model through enhanced uncoupled respiration via uncoupling protein (UCP) 2 and UCP 3 expression. Arcari *et al.* [49] also showed that yerba maté modulates thermogenesis by increasing the mRNA levels of *Pgc1α* (peroxisome proliferator-activated receptor gamma, coactivator 1 alpha) and UCP 1 in brown adipose tissue.

As mentioned before, obesity is associated with a state of chronic, low-grade inflammation characterized by abnormal cytokine production and the activation of inflammatory signaling pathways in adipose tissue [76]. Thus, the anti-inflammatory role of yerba maté has also been targeted. In adipose tissue, it has been shown that yerba maté has potent anti-inflammatory effects, downregulating the expression of *Tnf-α, Il-6, Lep* (leptin),* Pai1* (plasminogen activator inhibitor type), *Ccl2* (chemokine (C-C motif) ligand2) and *Ccr2* (chemokine (C-C motif) receptor 2) and upregulating *AdipoR1* (adiponectin receptor 1) [49]. In liver, yerba maté was found to reduce the nuclear translocation of NF-κB, which downregulates the mRNA levels of *Il-6, Nos2* (nitric oxide synthase 2) and *Tnf-α* [45]. Subsequently, it was observed that yerba maté reversed hypothalamic inflammation caused by high-fat diet by reducing IKK phosphorylation and NFκBp65 expression and increasing IκBα phosphorylation and the expression of Adipor1 and IRS-2 in the hypothalamus [46]. On the other hand, it was reported that yerba maté consumption did not affect the NF-κB signaling pathway in peritoneal macrophages; however, yerba maté consumption improved systemic markers of inflammation, such as IL-6, PAI1 and TNF-α [48].

**Figure 2:**
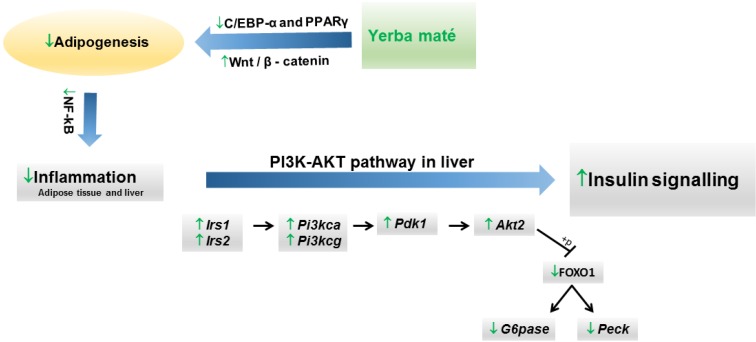
Proposed mechanism of action of yerba maté on the modulation of the PI3K-AKT signaling pathway.

It is well known that an increase in adipokine production can influence glucose metabolism, insulin sensitivity and inflammation, and this finding could represent a molecular link between obesity and the development of diabetes mellitus, metabolic syndromes and cardiovascular diseases [77]. The effects of yerba maté extract on insulin resistance and gene expression of inflammatory markers have been studied in animal models. Several studies indicate that yerba maté improves glucose tolerance in obese animals [43,45,48,57], along with an increase in the constant of insulin tolerance test (KITT) value [45,57]. In addition, yerba maté inhibited hepatic *Tnf-α* and restored hepatic and muscle insulin signaling through an increase in IRS-1 tyrosine phosphorylation in mice with high fat diet-induced obesity [45]. Because one of the earliest steps in the insulin signaling pathway is phosphatidylinositol 3-kinase (PI3K) activation [78], the effects of yerba maté on this pathway have also been reported. Arcari *et al*. [57] demonstrated that yerba maté has a modulatory effect on different insulin-related target genes (*Akt2, Irs1, Irs2, Pi3kca, Pi3kcg* and *Pdk1* (pyruvate dehydrogenase kinase, isoenzyme 1)) in the liver of animals subjected to a high-fat diet. Furthermore, yerba maté downregulates phosphoenolpyruvate carboxykinase 1, cytosolic (*Pepck*) and glucose-6-phosphatase, catalytic (*G6p*c), the main gluconeogenesis genes, through a decrease in forkhead box O (FOXO) 1 nuclear translocation (Figure 2).

## 4. Conclusions

In summary, the data presented here showed that the use of yerba maté might be useful against obesity, improving the lipid parameters in humans and animal models. In addition, yerba maté modulates the expression of genes that are changed in the obese state and restores them to more normal levels of expression. In doing so, it addresses several of the abnormal and disease-causing factors associated with obesity. Protective and ameliorative effects on insulin resistance were also observed. Thus, as a general conclusion, it seems that yerba maté beverages and supplements might be helpful in the battle against obesity.
